# An agent-based model reveals lost person behavior based on data from wilderness search and rescue

**DOI:** 10.1038/s41598-022-09502-4

**Published:** 2022-04-07

**Authors:** Amanda Hashimoto, Larkin Heintzman, Robert Koester, Nicole Abaid

**Affiliations:** 1 Engineering Mechanics Program, Virginia Polytechnic Institute and State University, Blacksburg, VA 24061 USA; 2 Department of Electrical and Computer Engineering, Virginia Polytechnic Institute and State University, Blacksburg, VA 24061 USA; 3 dbS Productions LLC, Charlottesville, VA 22911 USA; 4 School of the Environment, Geography and Geosciences, University of Portsmouth, Portsmouth, P01 2UP UK; 5 Department of Mathematics, Virginia Polytechnic Institute and State University, Blacksburg, VA 24061 USA

**Keywords:** Human behaviour, Computational models

## Abstract

Thousands of people are reported lost in the wilderness in the United States every year and locating these missing individuals as rapidly as possible depends on coordinated search and rescue (SAR) operations. As time passes, the search area grows, survival rate decreases, and searchers are faced with an increasingly daunting task of searching large areas in a short amount of time. To optimize the search process, mathematical models of lost person behavior with respect to landscape can be used in conjunction with current SAR practices. In this paper, we introduce an agent-based model of lost person behavior which allows agents to move on known landscapes with behavior defined as independent realizations of a random variable. The behavior random variable selects from a distribution of six known lost person reorientation strategies to simulate the agent’s trajectory. We systematically simulate a range of possible behavior distributions and find a best-fit behavioral profile for a hiker with the International Search and Rescue Incident Database. We validate these results with a leave-one-out analysis. This work represents the first time-discrete model of lost person dynamics validated with data from real SAR incidents and has the potential to improve current methods for wilderness SAR.

## Introduction

Between 2004 and 2014, in the US National Parks alone, there were 46,609 individuals who became lost and required a search and rescue campaign, which cost about 51.4 million dollars in total^1^. Wilderness search and rescue (SAR) not only consumes many hours and monetary expenses for searchers annually, but it also comes at a great cost to the searchers involved, both physically and mentally. These searches can involve great risks to human searchers due to the pressure from having to search large areas under strict time constraints, as time impacts chances of a lost person’s survival. However, coordinated SAR operations are the only way to help locate a missing individual alive. Therefore, it is of critical importance to make these searches as efficient as possible by utilizing methods to better understand lost person behavior.

A lost person (LP) is defined as a person unable to identify or orient themselves with respect to known locations and with no effective means or method for reorientation^2^. In this definition, there are two parts: confusion in identifying current location and an inability to orient. This lack of ability to reorient oneself drives LPs to use a variety of different behaviors. In *Lost Person Behavior*, Robert Koester has defined behavior strategies that have been reported by LPs through the collection of incident data^3,4^. Different types of LPs, differentiated by demographics such as age, cognitive or emotional state, and activity performed prior to being lost, are prone to specific reorientation behaviors^2^. A hiker, for example, may rely on aids such as roads and trails for travel, while a person with dementia may travel in one direction regardless of the terrain^3,5^. The psychological effect of being lost and behaviors associated with it have been studied extensively by environmental psychologists^6–8^. In this work, we use these behavioral profiles, or lost person types (LPTs), which the SAR community refers to as Subject Categories, to inform our model of lost person dynamics in the wilderness.

As it is currently practiced, a SAR team will initially create a probability distribution map of likely locations of the LP based on terrain features, the profile of the LP, weather, and input from SAR experts^3^. Searchers must know the initial location of the LP, whether it is the point last seen by an eyewitness, or the last known point where there is substantial evidence to place the lost person. In either case, this point is called the initial planning point (IPP) and is used to measure the progress of the search. The incident commander will allocate resources and coordinate searchers based on the information available and the probability map^9^. Good planning and efficient task allocation in the first few hours of the search can make a significant difference to the success of the search. As time progresses, the survivability of the lost person decreases rapidly, especially so if the LP is not found within the first 51 hours; in other words, effective planning can mean life or death^10^. Thus, the quality of the predicted locations of the LP is critical to search operations. Currently, these predictions are based on heuristics employed by the incident commander, which may not be able to simultaneously take into account geophysical and transient landscape features, and demographic and activity information about the LP. The goal of this article is to introduce a novel dynamic model of lost person behavior which synthesizes information about the specific environment for a search, as well as characteristics of the LP drawn from a large database of search incidents.

The International Search and Rescue Incident Database (ISRID), created and curated by Koester and reported in *Lost Person Behavior*, has data from more than 145,000 searches from around the world and identifies strategies lost people may use^3,11,12^. The book identifies more than 30 categories of LPs, based on activity or demographic information. The reported metrics include the horizontal and vertical distance found from the IPP, the time the lost person remained mobile while lost, and the status when found. The summarized metric statistics are distinct for each lost person category. In practice, searchers use these statistics to create *ad hoc* probability maps, like a distance-ring model^5^, in order to predict the location of the LP. However, these tactics assume that, by the time the search has started, the lost person has stopped moving^13^. As the community seeks to streamline SAR operations, search teams are deployed faster and the assumption that the LP is static no longer holds. Therefore, motion should be considered in the planning phase in order to create a probability map that evolves in time.

There are existing models in the literature on lost person dynamics in the wilderness. Many existing models of human behavior have been used mainly to study pedestrian dynamics, including force-based models showing collision avoidance, vision-based guidance, or goal-oriented behaviors^14–19^ and agent-based models based on behavioral heuristics^20–23^. An LP model is fundamentally different from pedestrian dynamics, since the behavior of the LP depends heavily on the landscape and the LP type, as evidenced by the statistics in ISRID. Models of LP behavior in the literature are both deterministic and stochastic and may account for or neglect the local landscape. The watershed model analyzed by Doke^24^ and described by Sava et al.^13^ embodies the idea that an LP will move on a path to minimize watershed crossings. The distance-ring model uses statistics from a database like ISRID to draw concentric circles to bound the LP’s possible position after a given time^3^, but neglects specific features of the landscape and only relies on the maximum distance an LP could potentially traverse. Also drawing from ISRID, McDaniel has created an agent-based model using behavior strategies and a detailed landscape environment^25^, which would be strengthened by using real search incidents over summarized statistics. Morelle et al. developed a spatially explicit agent-based model to investigate the movement of individuals at the interface of urban and natural settings^26^. While this model depends on the landscape, the model was only simulated in and around a midsized town environment and does not include time. A Bayesian model created by Lin and Goodrich only considers terrain, but not strategies or lost person types^27^. Mohibullah has expanded the Bayesian model^27^ and created an agent-based model using different strategies which also accounts for the fact that the LP has an internal state that evolves over time as it is moving, that is, the LP can become fatigued^28^. In order to evaluate the model, the authors compared the simulated tracks to actual recordings of participant movement in a wilderness environment^29^. Though motion tracks are desirable, a controlled experiment can lack the behaviors expressed from the true psychological effects of being lost, and an evaluation using genuine search incidents is preferable. Alanis et al. created a mechanistic model for a lost hiker that evaluates the influence of a simulated terrain in combination with lost person behavior using a finite time horizon Markov Decision Process^30^. Though it incorporates both geographic information and behavioral analysis, the model lacks the use of real map data and actual search incidents. In contrast, Šerić et al. use real search incidents in a cellular automata-based algorithm to determine the search area for a lost person using walking speed as the main parameter^31^. Their results show that landscape features play a large role in LP movement and should always be considered when planning a search area. Another model by Metcalfe uses a probability-based movement within a grid and emphasizes the importance of topography and fatigue on lost person movement^32^. However, in both of these cases, the authors neglect lost person strategies. Considering previous literature, the gap in current knowledge becomes clear. In this paper, we propose a model that incorporates different lost person types, known behavior strategies, and a detailed landscape environment that is evaluated using a large database of lost person incidents.

We seek to use a different approach from the previous work on LP behavior which incorporates the idea that LPs can be of the different types defined in *Lost Person Behavior*^3^, and which generates specific routes taken by the LP on a known landscape. This approach gives an expectation for the LP location at an arbitrarily high spatiotemporal resolution. This work expands on a zeroth-generation version of the model^33^ to include all salient behavior strategies and updated terrain features, and we evaluate the results using an LP incident dataset instead of the summarized statistics from ISRID. Through simulation of all possible distributions of behaviors, we are able to compare the lost person types (LPTs) taken from real-world data to the behaviors used in our model and validate the profiles against information from ISRID. We anticipate that this model, since it generates potential trajectories of an LP on the landscape, will facilitate SAR efforts as they currently are, as well as enable new SAR practices such as the use of UAV teams to search large areas of land more efficiently.

The organization of this paper is as follows. We begin by describing the model, including map generation, lost person behavior strategies, and behavioral profiles. Next, we outline the simulations, detailing the dataset used to fit the model, simulation parameters, and the metric to validate the model fitting. The results of the simulations are detailed next, followed by the discussion and conclusions.

## Modeling

In this section, we describe how we generate the maps on which lost person dynamics are simulated, then we define the behavior strategies an LP may use and show how these strategies combine to make a particular lost person type.

### Map

The LP is modeled as a self-propelled agent moving in discrete time on a two-dimensional square grid of fixed side length. The grid represents a specific map region and each cell is informed by the location’s geophysical characteristics. These specific characteristics are pulled from USGS geographic information system (GIS) data, which provides map layers at a given resolution, and are used in this work to define how the LP interacts with the environment.

Maps comprise two types of features: (1) linear features that an LP may follow and (2) inaccessible areas where an LP cannot traverse. When lost, it is common to follow predefined paths, like hiking trails, roads, railroads, powerline easements, and water features, and these one-dimensional routes are defined as *linear features* in the model. In addition to the structural linear features, we include paths defined by the elevation (with respect to sea level), like mountain crests and drainages. These features manifest as critical points in the magnitude of the gradient of elevation. After the gradient field is computed using the derivative of a Gaussian filter, the magnitude is computed and smoothed, and linear features are found using the Canny Edge Detection method by looking for local maxima and minima of a scalar field^34^. The Supplementary Information contains details on implementing these data treatments.

**Figure 1:**
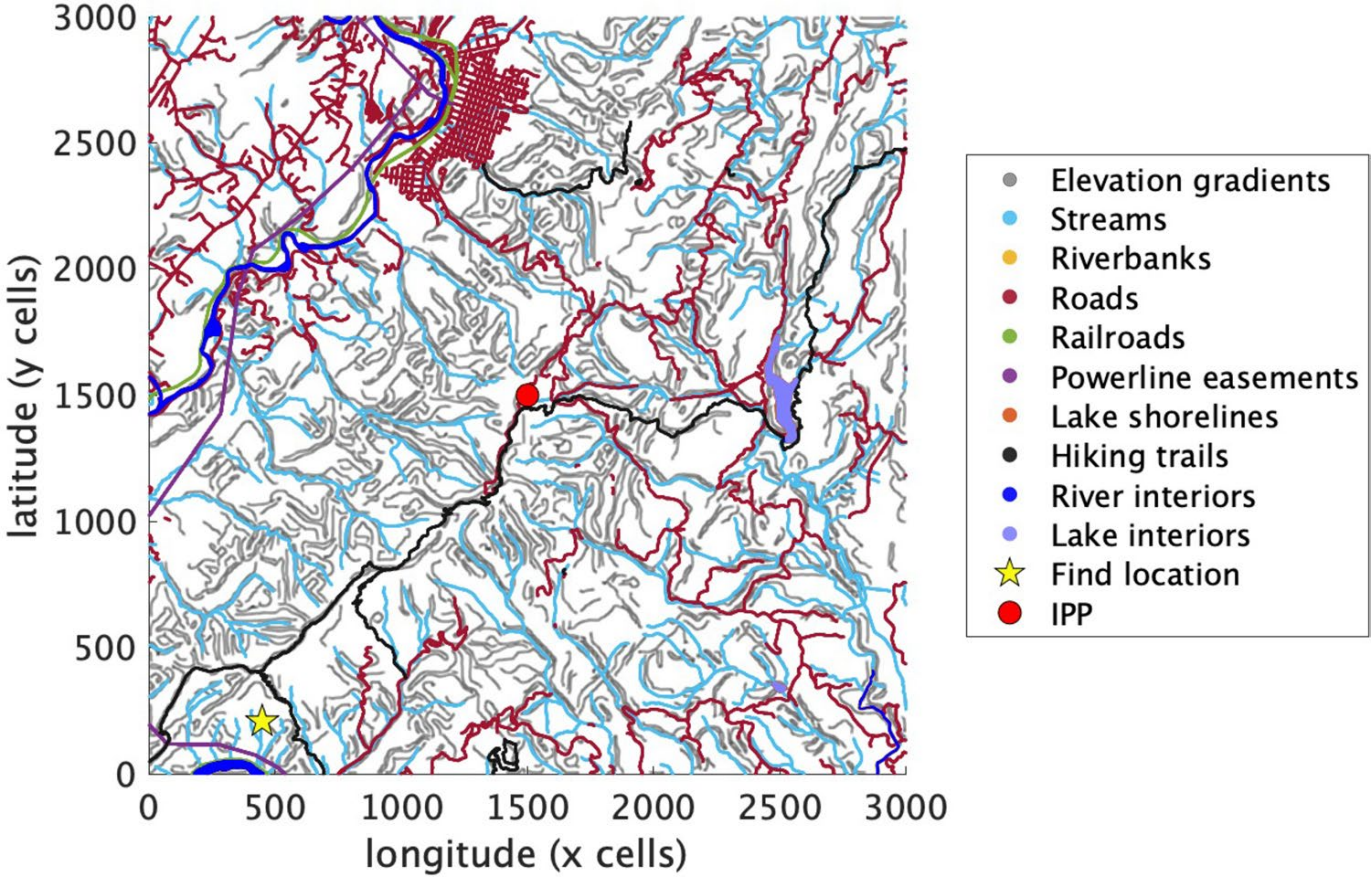
Example map of LP location with salient features. An example of an incident with its IPP (red circle), its corresponding find location in the lower left (yellow star), linear features (elevation gradients, streams, riverbanks, roads, railroads, powerline easements, lake shorelines, and hiking trails), and inaccessible areas (river and lake interiors). The map was generated using MATLAB 2020b and further details are included in the Supplementary Information.

In addition to linear features, the map contains *inaccessible areas*, specifically the interiors of lakes and wider rivers. By finding the boundaries of these water regions, we can separate the river and lake shorelines from their interiors and into the linear feature and inaccessible maps, respectively. In Fig. 1, an example of a map is shown with all linear feature and inaccessibility layers. As the agent moves on the grid, it will cross check its position with the feature map to make sure to avoid inaccessible areas and to use linear features depending upon its selected behavior strategy. Sufficiently steep terrain would also be considered an inaccessible area in theory, but this has not been implemented in the current version of the model.

### Lost person behavior strategies

The model defines how the LP moves between cells on the spatially discrete map. At every time step, the agent can move from its position to any of the eight adjacent cells, or stay in its current cell, by using an algorithm that defines possible behavior strategies. In the current model, we have defined six behaviors that are derived from *Lost Person Behavior*^3^:

1. *Random Walking* (RW) An agent moves randomly.
2. *Route Traveling* (RT) An agent travels on a linear feature.
3. *Direction Traveling* (DT) An agent moves cross-country in one compass direction, often ignoring trails and paths.
4. *Staying Put* (SP) An agent actively stays in the same location.
5. *View Enhancing* (VE) An agent attempts to gain a position of height.
6. *Backtracking* (BT) An agent attempts to follow the exact route traveled previously.

Each lost person type or LPT is defined by a probability mass function (PMF) that captures the probability of the agent using a specific strategy at each time step. The PMF is a six-element vector of probabilities of each of the above strategies which sums to one. For example, an LPT with a probability of [RW, RT, DT, SP, VE, BT] = [$\frac{1}{2}$, 0, $\frac{1}{6}$, $\frac{1}{3}$, 0, 0] has a 50% chance of random walking, a 17% chance of direction traveling, and a 33% chance of staying put at a given time step. Independent realizations of this distribution of behaviors are generated at each time step and the agent’s position is updated by the randomly selected strategy.

**Figure 2:**
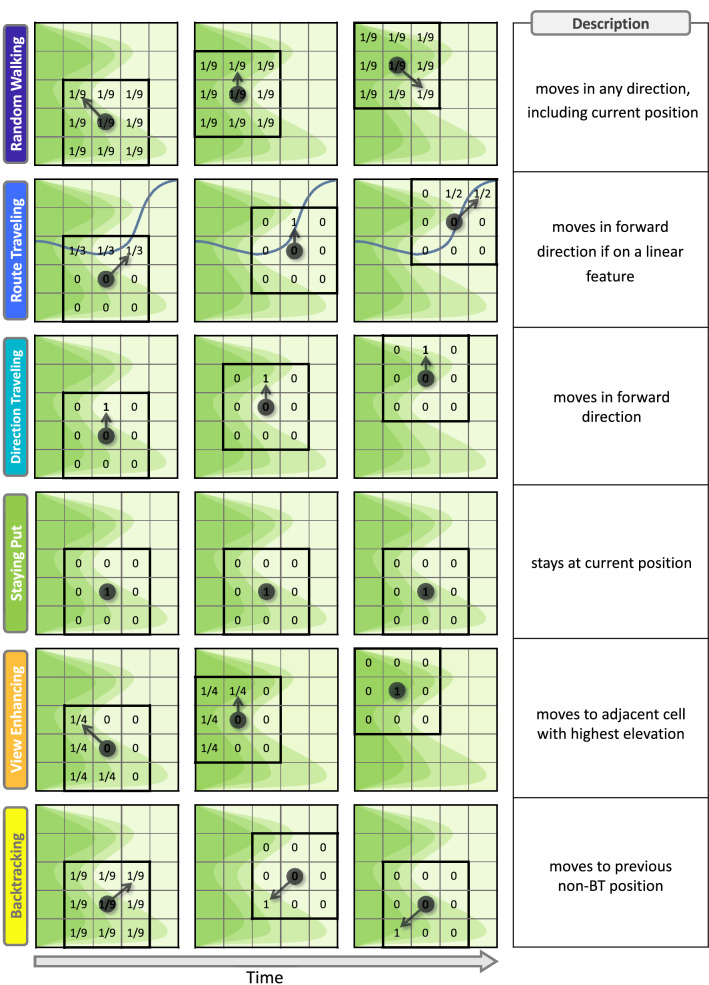
Schematic of an agent’s trajectories for all six behavior strategies over time. The agent’s initial positions are the circles in the left columns, located in the center of the bolded $3 \times 3$ grids, with the arrows denoting their next moves. The number in each box is the probability of updating to that position in the next time step using the strategy in each row, with time increasing by one step from left to right. All boxes with no number have zero probability. Linear features are represented as the blue line in the route traveling strategy, and elevation increases with a darker green contour.

To begin a simulation, the initial position of the agent $x(1)\in \mathbb {N}^2$ is selected and the second position $x(2)\in \mathbb {N}^2$ is randomly generated from the eight adjacent cells relative to *x*(1). The initial velocity is computed as $v(1) = x(2)-x(1)$. The difference between successive positions can be thought of as a velocity, which facilitates implementing some behaviors that rely on the direction of motion, like direction traveling. When the position is updated at each time step, we use a smoothing factor $\alpha$ to take into account the previous velocity and make the trajectories of similar smoothness to a walking individual by introducing one time step of memory. The updated smoothed position at each time step *t* is computed as

1 $$\begin{aligned} x(t+1) = (2-\alpha )x(t) + (\alpha -1)x(t-1)+\alpha v(t) \end{aligned}$$

where $v(t)=\hat{x}(t+1)-x(t)$ and $\hat{x}(t+1)$ is the provisional update for the behavior strategy selected. At each time step *t*, an independent realization of a selected PMF is generated, defining the strategy the agent will use for the updated position $x(t+1)$ and velocity $v(t+1)$. Placing the agent at the center of a $3\times 3$ grid that is a subset of the larger discrete map, in body coordinates local to the agent’s position, we generate motion by selection of one of the six strategies. At time step *t*, the $3\times 3$ grid (seen in Fig. 2), with *x*(*t*) at its center is positioned so that it aligns with the velocity *v*(*t*). On the square grid of the map, this is accomplished by appropriately rounding the coordinate values when this orientation is not orthogonal to the global axes.

The six strategies above define updates for the position of the agent with respect to the $3\times 3$ grid and $x(t+1)$ in global coordinates. The strategies and their respective PMFs are shown in the schematic in Fig. 2. When the individual walks randomly (RW), the chance of moving into any adjacent cells, including its own, is the same. When the agent is route traveling (RT), it checks each of the surrounding cells for a linear feature. Then the updated position is randomly selected (with a uniform probability) from the at most three possible positions in the direction of motion in body coordinates if a linear feature is present. This definition enforces persistence in the direction of motion along a linear feature. If a feature is not present, the agent performs a random walk. When the individual uses direction traveling (DT), the agent only moves forward in body coordinates. The previous direction of travel is taken into account by the orientation of body coordinates parallel to the velocity. When staying put (SP), the only possible update is the agent’s previous location. When view enhancing (VE), the agent checks the elevation for each of the adjacent cells against its own current elevation and selects the cell with the highest altitude. If it is currently at the highest elevation, the agent stays put. Lastly, when backtracking (BT), the previous behavior is first checked to see if it was also BT, and then, if it is not, the updated position is the previous location. If the previous behavior was backtracking, the agent uses the last non-BT position. In this strategy, the agent is following its path backwards to previous positions. In the case that the agent tries to move into an inaccessible area, it stays put for the current time step regardless of the behavior used.

Then, the model is iterated for *T* time steps to generate the agent’s trajectory.

### Behavioral profiles

**Figure 3:**
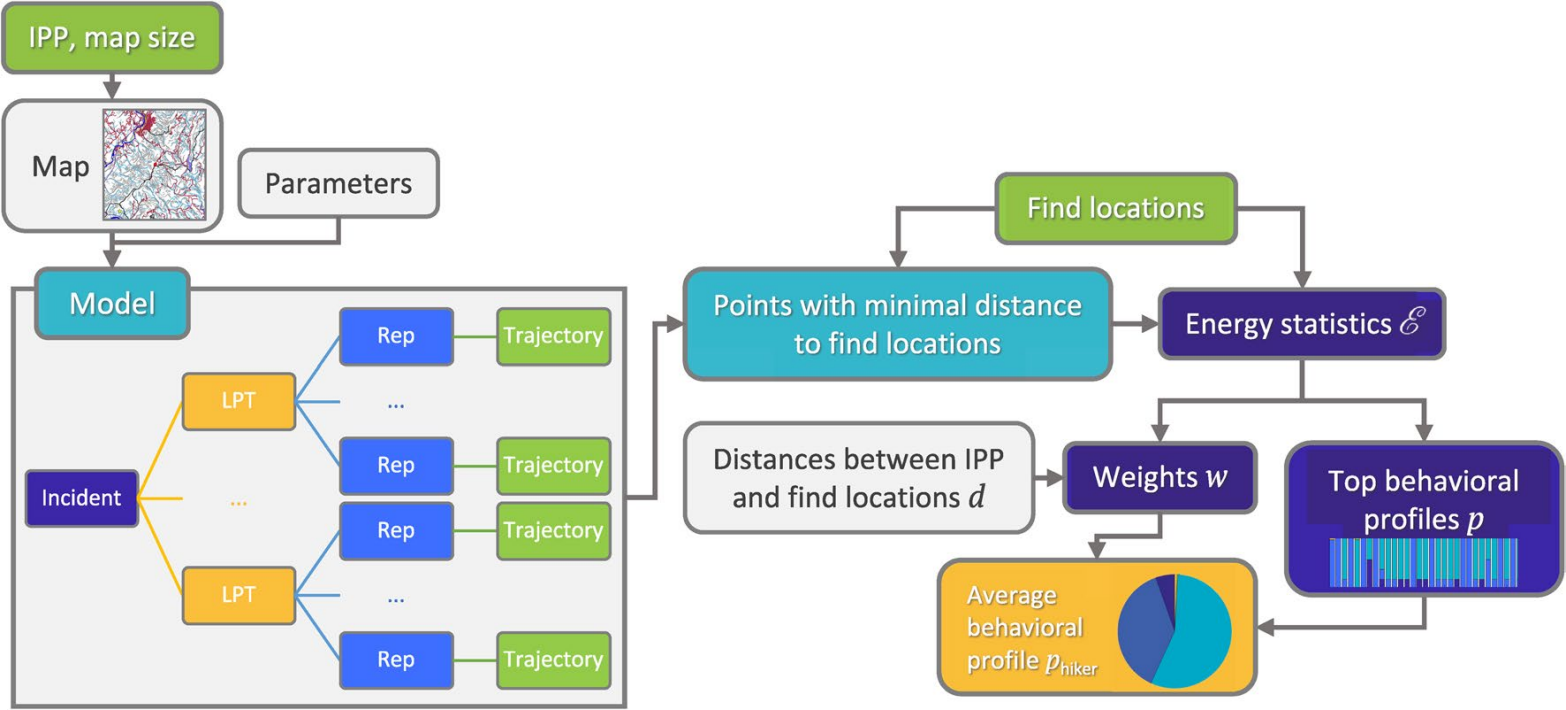
Flowchart of the lost person model algorithm.

The lost person model can simulate a multitude of lost person types, including child, hunter, or hiker, to name a few. In order to demonstrate the power of the model, we have chosen to only simulate a hiker lost person because of the large amount of data available and its prevalence in SAR incidents. To determine the behavioral profile for a hiker, we generate all possible permutations of the six behavior strategies as LPT PMFs, with the probability of each behavior as a multiple of $\frac{1}{6}$. Incrementing the probability of each strategy from zero to one by steps of $\frac{1}{6}$ and retaining only the distributions that sum to one, we have a set of 462 LPTs with varying proportions of each behavior. Each of these LPT distributions are simulated for 500 Monte Carlo replicates for each incident. A flowchart detailing the model algorithm is in Fig. 3.

## Simulations

In this section, we describe the SAR incident data used to fit the model, we detail simulation parameters, and we define a metric for comparing numerical results.

### LP incident data

For the simulation study, we use an augmented data set of lost person incidents from ISRID that includes a wealth of information, like the demographic information about the LP, number of lost people in an incident, region, and find location. The data used in this study comes from a collection of pre-existing lost person incidents and are thus retrospective. In particular, our simulations rely on the initial planning points (IPPs), find locations, and LP types, where the IPPs and find locations are defined as the simulations’ initial conditions and end criteria, respectively. Then, the LP type that minimizes differences with the real find locations is identified. We select a subset of the hiker incidents using exclusion criteria for the find location. Specifically, we exclude locations where the find location is within 1 km of the IPP, outside the simulated map limits, in an inaccessible area as defined by our GIS layers, or within 100 cells of the map boundary. After applying these criteria, we have a set of $N=65$ incidents on which to simulate our model that range across the United States. Each lost person incident is defined with GPS positions for both an IPP and a find location. The dataset used for this study is included in the Supplementary files. As a note, this data appears among the many incidents summarized by the statistics in publicly available datasets^3,12^.

For each of the incidents, a $20\,\mathrm {km} \times 20 \,\mathrm{km}$ map is generated with its corresponding layers for elevation, linear features, and inaccessible areas, with the IPP placed at the center as the initial position. The map is discretized into a $3000 \times 3000$ cell grid, where cells are $6.67\,\mathrm {m} \times 6.67\,\mathrm {m}$ squares, and the IPP is at $(x,y)=(1500,1500)$. Figure 1 is an example of one incident’s map, showing all of the linear features and the inaccessible areas.

### Simulation parameters

The maps are generated using Python v3.6.8 and the elevation and layer data are derived from ArcGIS services^35^ using AGS Tools^36^. The simulations are performed using MATLAB 2020a on the Virginia Tech Advanced Research Computing Cascades cluster^37^. The complete list of parameters is in Table 1. The simulation lengths are set for $K=100$ hours, where each time step is based on a maximum walking speed of about 1.575 meters per second. This corresponds to $T=850$ time steps per hour, where *T* is multiplied by the length *K* to define the lengths of each Monte Carlo replicate. The choice of time step is determined by the average walking speed of a human, which is about 3.5 miles per hour, or 1.56 meters per second^38^. By simulating for $K=100$ hours, the agent has the opportunity to traverse the entire map. The selection of the smoothing factor, $\alpha$, is to generate trajectories that are qualitatively as smooth as known trajectories from real hikers. Moreover, the value of $\alpha = 0.55$ is selected in particular to avoid cancellation in variables that may happen when a fair weighting of $\alpha =0.5$ is used. We simulate each of the $N=65$ incidents for 462 LPTs with 500 replicates.

**Table 1:** Simulation parameters.

Variable	Symbol	Value
Smoothing parameter	$\alpha$	0.55
Simulation length in hours	*K*	100
Simulation time steps per hour	*T*	850
Monte Carlo replicates	–	500
Number of incidents	*N*	65

### Energy statistic

To explore the validity of the model, we use a statistic to compare the simulated trajectories to the actual find locations for each of the incidents. Energy distance is a metric which quantifies the statistical distance between distributions of random vectors, thereby characterizing equality of distributions^39,40^. To test for equal distributions, we consider the null hypothesis that two random variables *X* and *Y* have the same probability distributions. For samples from *X* and *Y*, $x_{1}, \ldots , x_{n}$ and $y_{1}, \ldots , y_{m},$ respectively, the energy statistic for testing this null hypothesis is defined as

2 $$\begin{aligned} \mathscr{E}(X, Y):=2 A-B-C \end{aligned}$$

where *A*, *B*, and *C* are the averages of pairwise distances between the *X* and *Y* samples:

3 $$\begin{aligned} A&=\frac{1}{n m} \sum _{i=1}^{n} \sum _{j=1}^{m}\left\| x_{i}-y_{j}\right\| ,&B&=\frac{1}{n^{2}} \sum _{i=1}^{n} \sum _{j=1}^{n}\left\| x_{i}-x_{j}\right\| ,&C&=\frac{1}{m^{2}} \sum _{i=1}^{m} \sum _{j=1}^{m}\left\| y_{i}-y_{j}\right\| . \end{aligned}$$

In our case, for each incident, we find the closest point of each replicate that minimizes the distance from the simulated trajectory to the actual find location and define this as realizations of *X* for each of the 462 behavior distributions. *Y* is the actual find location given from the incident dataset, which is a single GPS point. In other words, $n=500$ and $m=1$. We calculate the pairwise distances between $X-Y$ and *X* to find *A* and *B*, and thus the energy statistic. Since *Y* only has a single realization, the value for *C* is zero.

**Figure 4:**
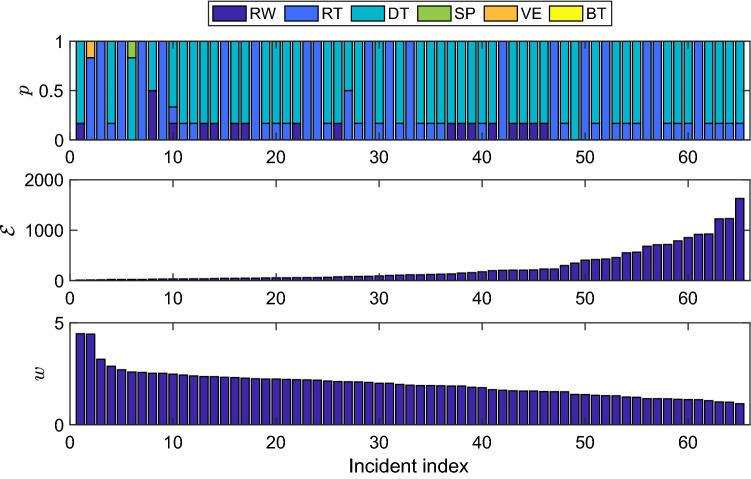
Results from model simulations and fitting. The behavioral profile *p* (top), energy statistic $\mathscr{E}$ (middle), and weight *w* (bottom) for all $N=65$ incidents sorted from highest to lowest weight.

We define the best behavior distribution for incident *i* as the LPT with the lowest energy statistic, giving us a top behavioral profile, $p_i$. For example, the behavioral profile for the nineteenth incident is $p_{19}=\left[\frac{1}{6},0,\frac{5}{6},0,0,0\right]$ which is a normalized distribution of the six behavior strategies [RW, RT, DT, SP, VE, BT]. As a way to synthesize these profiles across all the incidents, we assign a weight $w_i$ to each probability distribution $p_i$ that is a power of the inverse of energy per unit distance, defined as

4 $$\begin{aligned} w_i = \bigg (\frac{d_i}{\mathscr{E}_i}\bigg )^L \end{aligned}$$

for $i=1,\ldots , N$. This quantity is based on the incident’s energy statistic $\mathscr{E}_i$, the distance $d_i$ measured between the IPP and the actual find location, and a characteristic length parameter *L*. We select $L=\frac{1}{2}$ to more heavily represent the better fitting distributions. In Fig. 4, the best fit behaviors *p* for each incident are shown in the top subfigure with the corresponding energy statistic $\mathscr{E}$ and weight *w* in the second and third subfigures, respectively. The incidents have been sorted by their weight in Eq. (4), from highest to lowest.

**Figure 5:**
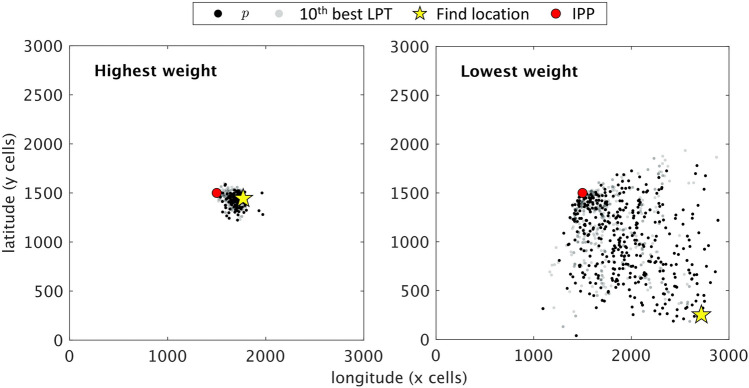
Examples of simulation results for two incidents. The highest (left) and lowest (right) weighted incidents with the trajectory closest points shown for *p* (black dots) and the tenth best LPT (grey dots) along with the IPP (red circle) and find location (yellow star).

**Figure 6:**
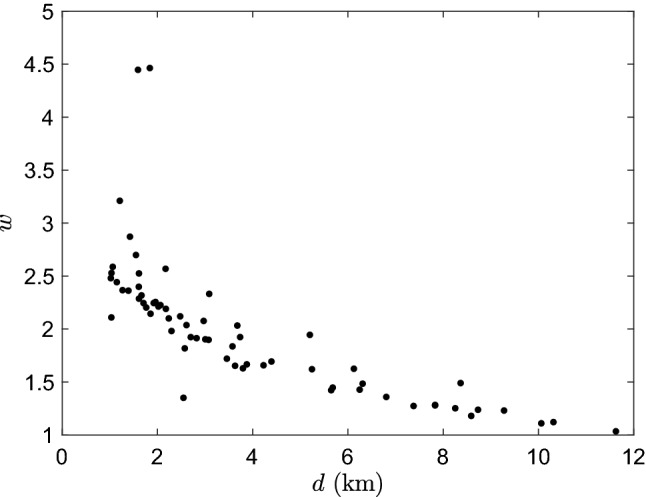
Comparison of best fit LPT weights versus distance for all incidents. The distance *d* in km is measured from IPP to find location and the corresponding weights *w* are for all $N=65$ incidents.

To give a better picture of how well a behavior fits an incident based on the corresponding energy distance, Fig. 5 shows incidents corresponding to the highest and lowest weights in Fig. 4. Each subfigure shows the closest points of the replicates’ trajectories for the first and tenth best fitting LPTs, the IPP, and find location. We expect to see the points from the first best LPT *p* (black dots) concentrated closer to the find location than the tenth best LPT (grey dots). The left plot of Fig. 5 is the highest weighted incident, and the distribution of points for *p* is closer to the find location than all other LPTs, including the other shown in the plot. On the other hand, the right plot of Fig. 5 is the lowest weighted incident and, while it can be seen that *p* is still the closest to the find location in comparison to the tenth, it is noticeable that the find location is substantially further from the IPP than in the left plot. It may be tempting to believe that the incidents with the shortest *d* would always be weighted higher, but this is not always the case. In Fig. 6, the distances and weights for all incidents are shown, and while there is a correlation between higher weights and shorter distances, deviations from this trend demonstrate the fact that the weight is defined inversely to the energy per unit distance and not merely energy.

## Results

### Average behavioral profile for a hiker

**Figure 7:**
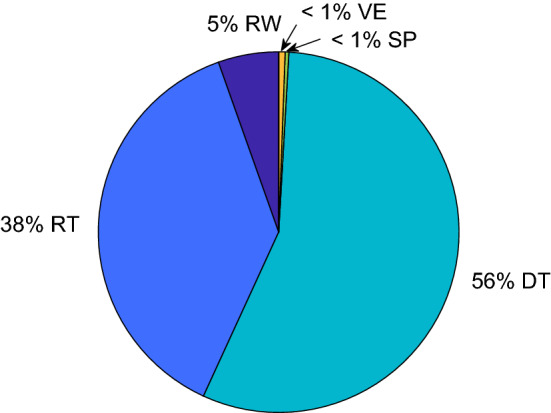
The average behavioral profile for a hiker, $p_{\mathrm {hiker}}$.

Once the best behavioral profile and associated weight has been identified for each incident, we can combine them to find the *average behavioral profile* for a hiker. Using these best fits, we compute the weighted average

5 $$\begin{aligned} p_{\mathrm {hiker}} = \frac{w_1 {p}_1+w_2 {p}_2+\cdots +w_N {p}_N}{|w_1 {p}_1+w_2 {p}_2+\cdots +w_N {p}_N|} \end{aligned}$$

where the distribution is normalized to sum to one and the weights are defined as in Eq. (4) for the $N=65$ incidents. The resulting distribution $p_{\mathrm {hiker}}$ corresponds to a behavioral profile of [RW, RT, DT, SP, VE, BT] $=[0.055, 0.377, 0.559, 0.003, 0.006, 0]$, and is shown in Fig. 7.

### Evaluation of fit by cross validation

This average behavioral hiker profile can be evaluated using statistical measures to determine the goodness of the fit with the experimental data. We evaluate the method of fitting the model using a statistical analysis called *leave-one-out cross-validation* (LOOCV)^41–43^. To perform cross validation, we partition all of the samples into two subsets, apply a numerical fitting predictor to one subset (the training set), and then assess its prediction performance by comparison against the other subset (the test set)^44^. In LOOCV, for a dataset consisting of *k* samples, a single observation is *left out* as the test set and the remaining $k-1$ observations make up the training set. For the evaluation of the lost person model, this means that the model is fit with a behavioral profile *N* times, each time leaving out one incident in the training set.

First, for each of the *N* incidents, we train a weighted average behavioral profile using the method in the previous section on the subset of $N-1$ incidents (training set), where the left out incident is the test set. Second, the model is simulated for 500 Monte Carlo replicates for each of the *N* incidents using the corresponding trained behavioral profile as its behavior (i.e. 1 LPT instead of the original 462). The output of the LOOCV is *N* energy statistic values that can be compared to the original energy statistics.

To show the validity of the model fitting, the energy statistics from the LOOCV would need to be statistically “better” than the original energy statistics from the “untrained” data. To see this, we calculate the percentile of the LOOCV energy statistics among the 462 original energy statistics. The results show that $58.5\%$ of the *N* IPPs have energy statistic values above the 95th percentile, and $98.5\%$ above the 50th percentile. This means that the average behavioral profile captures very well the data from more than half of the SAR incidents on which it was not trained and works well across all the incidents in general.

## Discussion

The model in this paper is designed to capture lost person dynamics, based on behavior heuristics, over a variety of landscapes using a real-world dataset. Based on the goodness of the leave-one-out analysis, the average behavioral profile represents a hiker well on a variety of landscapes. Specifically, the weighted average behavioral profile offers a very good fit to more than half of the incidents and it performs better than most behavioral profiles for almost all incidents. We stress that this analysis tests the ability of the model to predict data on which it was not trained. Thus, the average behavioral profile provides a description of the LP behavior which no longer depends on any particular landscape features. This suggests that such a profile, which also could be similarly generated for LPs in other categories like children or people with dementia, could be tapped during active searches to predict areas where a lost person is more likely to be found.

Relating our findings to the real world, the values of the average behavioral profile $p_\mathrm {hiker}$ are consistent with what we know about hiker behavior. Hikers are likely to use behavior strategies that allow them to travel further distances and are often influenced by the surrounding landscape^30^. It has been seen that a third of lost hikers will move to a higher elevation to improve their view^11^, but many would prefer a clear path like a trail versus a steep one^24^. Our model hiker profile reflects some of these common behaviors. The best fit profile contains over $50\%$ direction traveling, which is the behavior that causes the agent to travel the furthest among the rest. The second most common behavior is route traveling, meaning the agent is utilizing the linear features of the landscape to navigate the terrain. Because of the somewhat serpentine trajectories by the simulated agents, we are not surprised to see that the most used strategies are route traveling and direction traveling. Out of all six behaviors, these strategies allow the agent to traverse the furthest on the map in order to reach the find locations.

**Figure 8:**
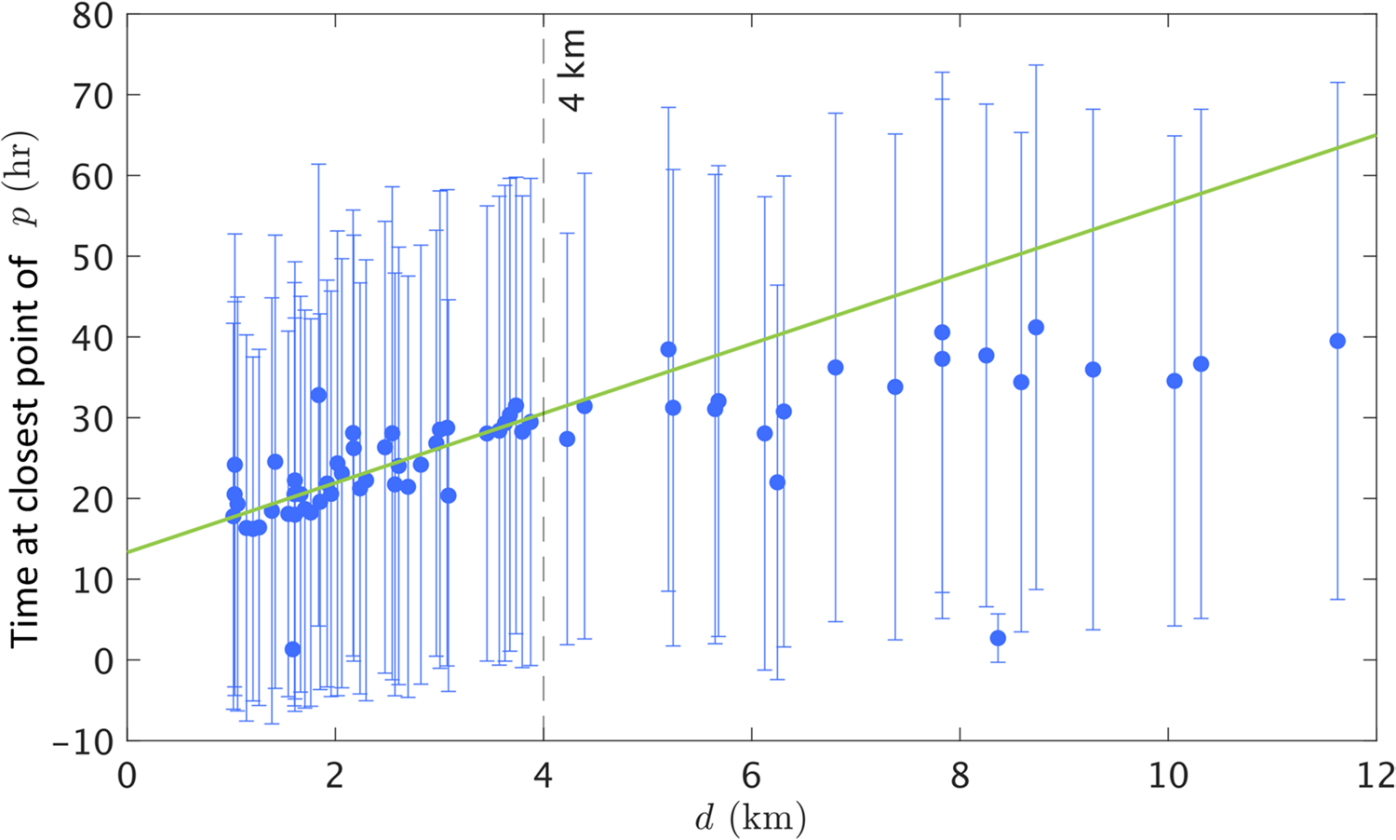
Time at trajectory closest points using *p* versus *d*. Circles and error bars show mean and one standard deviation over 500 replicates, respectively. The green line shows the linear fit, $t=4.31d+13.31$, for means with *d* less than 4 km, and its associated effective speed is computed as $0.064\,\mathrm {m/s}$.

Beyond the average hiker profile, the model results generate an effective agent walking speed by comparison of the time of the closest point for each *p* and the distance *d* from IPP to find location. In Fig. 8, the mean time (averaged over replicates) versus distance is shown for each incident. We notice that the data is dense and seems to be linearly increasing for distances less than 4 km; for distances greater than 4 km, the data is sparser and seems relatively constant. With these two regimes in mind, we fit the data with incidents with *d* less than 4 km using the MATLAB Curve Fitting Toolbox, and find a linear fit, $t=4.31d+13.31$, with $R^2=0.4383$. The slope of the line is used to compute the resulting *effective speed* of the LP model, which is $0.064\,\mathrm {m/s}$. Note that, compared to the maximum walking speed of $1.575\,\mathrm {m/s}$ which is biomechanically realistic for pedestrians^45^, the effective speed is very slow. This is not entirely surprising, due to the convoluted nature of the simulated agent’s paths, and we hypothesize that the speed would increase by including more memory into each updated time step by way of inertia. The relatively constant time to closest point for incidents with larger values of *d* suggests that the underlying dataset captures fatigue in the lost person. Since the time values saturate at approximately 35 hours, we expect that there is a maximum amount of time that a lost person remains mobile. This idea is supported by the ISRID, and many models of mobility attempt to estimate the distance traveled based on the time and energy it takes to navigate a terrain. Specifically, Tobler estimated travel speed to be a function of pedestrian movement and the slope of the landscape^46^. These cost-distance methods used in conjunction with the impact of land cover impedance have been shown to provide guidance in determining probability of area, but ultimately, they neglect the behavior of a lost person^47^. We hope to explore mobility in expansions of this model.

Although these results give a strong indication that the methods we use generate a good representation of lost person behavior, limitations arise from using recorded SAR incident data from ISRID. First of all, lost person data is difficult to attain as many incidents go unreported, since most of the people who get lost end up reorienting themselves. This means that certain types of lost people may be over-represented in the data. In the incidents that are recorded, actual trajectories of the LP are rare since the LP is unlikely to have a tracking device. By having real trajectories, we could fit the model to these tracks instead of just the IPP and find locations. The incidents in the dataset also lack a sense of time. In *Lost Person Behavior*^3^, the LP categories have a statistic called mobility to describe the amount of time an LP was mobile, but this variable is often artificially shorter than in reality since many LPs are still mobile when found. Because of this, we decided to run the model for an extended time and use the closest point in the trajectory for fitting in our analysis. The lack of time in the incident data also means that the map layers we use may not always reflect the same terrain as at the time of the incident. Furthermore, the model neglects land cover and vision restriction, which are both known to affect navigation as well as walking speeds^47^. Lastly, profiles for other LPTs have sparser data than hikers, which limits the implementation of the simulation and fitting method described in this paper.

## Conclusions

This study describes the first dynamic model of lost person behavior that takes into account known reorientation strategies and is informed by data from real SAR incidents. This model is unique from others, not only for the use of SAR incident data, but also for incorporating both landscape features and behavior strategies. Our results show, through a leave-one-out analysis, that the average behavior profile we generate for a hiker captures well the features of SAR incidents on which it was not trained. The model here provides a solid foundation for future work. We can imagine many expansions, including adding more types of LPs and implementing a more realistic effective speed of the agent. By incorporating memory of more than one previous time step, the trajectories would be smoother and perhaps more realistic to a walking individual. Furthermore, instead of a constant walking speed, we can introduce a variable speed depending on the terrain and LPT. Another aspect to consider in future work is the specific demographics of the LP, including age, sex, and whether the agent was alone or in a group. Weather also plays a large part in where an LP will be found. This work offers the first step in defining dynamic models for lost people which may be tailored to facilitate current state-of-the-art SAR efforts.

## Supplementary Information

Supplementary Information 1.

Supplementary Information 2.

Supplementary Information 3.

Supplementary Information 4.

## Data Availability

Data generated for this study are included as Supplementary Information files.

## References

[1] Federal Bureau of Investigation (FBI). 2018 NCIC Missing Person and Unidentified Person Statistics. Tech. Rep., National Crime Information Center (2018).

[2] Hill K (1998). The psychology of lost. Lost Pers. Behav..

[3] Koester, R. J. *Lost Person Behavior: A Search and Rescue Guide on Where to Look—for Land, Air and Water* (dbS Productions LLC, 2008).

[4] Hill KA (2012). Cognition in the woods: Biases in probability judgments by search and rescue planners. Judgm. Decis. Mak..

[5] Syrotuck, W. Analysis of lost person behavior. Barkleigh Productions. *Inc., Mechanicsburg, PA. NASAR version* (2000).

[6] Heth CD, Cornell EH (1998). Characteristics of travel by persons lost in Albertan wilderness areas. J. Environ. Psychol..

[7] Cornell EH, Heth CD, Broda LS (1989). Children’s wayfinding: Response to instructions to use environmental landmarks. Dev. Psychol..

[8] Cornell EH, Heth CD, Alberts DM (1994). Place recognition and way finding by children and adults. Mem. Cognit..

[9] Koester, R. J., Cooper, D., Frost, J. & Robe, R. Sweep width estimation for ground search and rescue. *United States Coast Guard National Search and Rescue Committee* 245 (2004).

[10] Adams AL (2007). Search is a time-critical event: When search and rescue missions may become futile. Wilderness Environ. Med..

[11] Koester, R. International Search and Rescue Incident Database (ISRID) (accessed 04 October 2019); http://www.dbs-sar.com/ (2008).

[12] Koester RJ (2020). Enhancements to statistical probability of area models based upon updated ISRID data collection for Autistic Spectrum Disorders and Typically Developing children. J. Search Rescue.

[13] Sava E, Twardy C, Koester R, Sonwalkar M (2016). Evaluating lost person behavior models. Trans. GIS.

[14] Seyfried A, Schadschneider A, Kemloh U, Chraibi M (2011). Force-based models of pedestrian dynamics. Netw. Heterog. Media.

[15] Moussaïd M, Helbing D, Theraulaz G (2011). How simple rules determine pedestrian behavior and crowd disasters. Proc. Natl. Acad. Sci..

[16] Fuchs, A. & Jirsa, V. K. *Coordination: Neural, Behavioral and Social Dynamics* (Springer, 2007).

[17] Karamouzas I, Skinner B, Guy SJ (2014). Universal power law governing pedestrian interactions. Phys. Rev. Lett..

[18] Seyfried A, Steffen B, Lippert T (2006). Basics of modelling the pedestrian flow. Phys. A Stat. Mech. Appl..

[19] Garcimartín A, Pastor JM, Martín-Gómez C, Parisi D, Zuriguel I (2017). Pedestrian collective motion in competitive room evacuation. Sci. Rep..

[20] Bonabeau E (2002). Agent-based modeling: Methods and techniques for simulating human systems. Proc. Natl. Acad. Sci..

[21] Takahashi, T., Tadokoro, S., Ohta, M. & Ito, N. Agent based approach in disaster rescue simulation - From test-bed of multiagent system to practical application. In *Lecture Notes in Computer Science (including subseries Lecture Notes in Artificial Intelligence and Lecture Notes in Bioinformatics)*, vol. 2377 LNAI, 102–111, 10.1007/3-540-45603-1_11 (Springer, 2002).

[22] Azimi, S., Delavar, M. R. & Rajabifard, A. Developing a multi-agent based modeling for smart search and rescue operation. In *Lecture Notes in Geoinformation and Cartography*, 133–157, 10.1007/978-3-030-05330-7_6 (Springer, 2019).

[23] Kratzke, T. M., Stone, L. D. & Frost, J. R. Search and rescue optimal planning system. In *2010 13th International Conference on Information Fusion*, 1–8, 10.1109/ICIF.2010.5712114 (IEEE, 2010).

[24] Doke, J. *Analysis of search incidents and lost person behavior in Yosemite National Park*. Master’s thesis, University of Kansas (2012).

[25] McDaniel, M. D. *Agent-based modeling of lost person wayfinding*. Master’s thesis, University of California Santa Barbara (2010).

[26] Morelle K, Buchecker M, Kienast F, Tobias S (2019). Nearby outdoor recreation modelling: An agent-based approach. Urban For. Urban Green..

[27] Lin L, Goodrich MA (2010). A Bayesian approach to modeling lost person behaviors based on terrain features in wilderness search and rescue. Comput. Math. Organ. Theory.

[28] Mohibullah, W. *Agent-based lost person movement modelling, prediction and search in wilderness*. Ph.D. thesis, UCL (University College London) (2017).

[29] Mohibullah, W. & Julier, S. J. Developing an agent model of a missing person in the wilderness. *Proceedings—2013 IEEE International Conference on Systems, Man, and Cybernetics, SMC 2013* 4462–4469, 10.1109/SMC.2013.759 (2013).

[30] Alanis, J. *et al.* Topography and behavior based movement modeling for missing hikers in land-wilderness settings. Tech. Rep., Quantitative Research for the Life and Social Sciences Program, Arizona State University (2019).

[31] Šerić L, Pinjušić T, Topić K, Blažević T (2021). Lost person search area prediction based on regression and transfer learning models. ISPRS Int. J. Geo-Inf..

[32] Metcalfe, M. *A probability-based lost person simulation for wilderness search and rescue operations*. Bachelor’s thesis, Boston University (2012).

[33] Hashimoto, A. & Abaid, N. An agent-based model of lost person dynamics for enabling wilderness search and rescue. In *Dynamic Systems and Control Conference*, vol. 59155, V002T13A005, 10.1115/DSCC2019-9222 (American Society of Mechanical Engineers, 2019).

[34] Canny, J. A computational approach to edge detection. *IEEE Transactions on Pattern Analysis and Machine Intelligence* **PAMI-8**, 679–698, 10.1109/TPAMI.1986.4767851 (1986). 21869365

[35] U.S. Geological Survey. USGS TNM National Hydrography Dataset (accessed 01 March 2021); https://hydro.nationalmap.gov/arcgis/rest/services (2021).

[36] Heintzman, L. ARCGIS Tools (accessed 01 March 2021); https://git.caslab.ece.vt.edu/hlarkin3/ags_grabber (2021).

[37] Virginia Tech Advanced Research Computing (ARC). Cascades Cluster (accessed 28 March 2021); https://198.82.212.30 (2021).

[38] Browning RC, Baker EA, Herron JA, Kram R (2006). Effects of obesity and sex on the energetic cost and preferred speed of walking. J. Appl. Physiol..

[39] Székely GJ, Rizzo ML (2013). Energy statistics: A class of statistics based on distances. J. Stat. Plan. Inference.

[40] Rizzo ML, Székely GJ (2016). Energy distance. Wiley Interdiscip. Rev. Comput. Stat..

[41] Lachenbruch PA, Mickey M Ray (1968). Estimation of error rates in discriminant analysis. Technometrics.

[42] Geisser S (1975). The predictive sample reuse method with applications. J. Am. Stat. Assoc..

[43] Drehmer DE, Morris GW (1981). Cross-validation with small samples: An algorithm for computing Gollob’s estimator. Educ. Psychol. Meas..

[44] Stone M (1974). Cross-validatory choice and assessment of statistical predictions. J. R. Stat. Soc. Ser. B (Methodol.).

[45] Bohannon RW (1997). Comfortable and maximum walking speed of adults aged 20–79 years: Reference values and determinants. Age Ageing.

[46] Tobler, W. *Non-isotropic geographic modeling* (University of California, Santa Barbara, Three presentations on geographic analysis and modeling. Tech. Rep., 1993).

[47] Doherty PJ, Guo Q, Doke J, Ferguson D (2014). An analysis of probability of area techniques for missing persons in Yosemite National Park. Appl. Geogr..

