# Supplementary material for: An agent-based model reveals lost person behavior based on data from wilderness search and rescue
Source: Sci Rep. 2022 Apr 7;12:5873. doi: 10.1038/s41598-022-09502-4 (PMC8990012; doi:10.1038/s41598-022-09502-4)
Supplement: Supplementary file 4 — Supplementary Information 4. [file 41598_2022_9502_MOESM4_ESM.pdf]

Supplementary Information:

An agent-based model reveals lost person behavior based on data from wilderness search and rescue

Amanda Hashimoto[1], Larkin Heintzman[2], Robert Koester[3,4], and Nicole Abaid[5,*]

[1] Virginia Polytechnic Institute and State University, Engineering Mechanics Program, Blacksburg, VA 24061, USA
[2] Virginia Polytechnic Institute and State University, Department of Electrical and Computer Engineering, Blacksburg, VA 24061, USA
[3] dbS Productions LLC, Charlottesville, VA 22903, USA
[4] University of Portsmouth, School of the Environment, Geography and Geosciences, Portsmouth, P01 2UP, UK
[5] Virginia Polytechnic Institute and State University, Department of Mathematics, Blacksburg, VA 24061, USA
* **Email:** nabaid@vt.edu

Map generation

The maps are created using Python v3.6.8 along with several common open-source packages, and elevation and layer data from ArcGIS. The map generation input is a GPS location at the center of the area of interest, an extent in meters which determines the size of the output matrix, and a set of ArcGIS feature layer URLs. The map generation output consists of two binary-valued matrices, one for linear features and one for inaccessibility, with each cell's value indicating the presence of a linear feature at that location or whether it is inaccessible, respectively. We separate the inaccessibility matrix from the linear feature matrix so that the boundaries of inaccessible areas can be used as linear features. In our case, the GPS locations are given by the 65 incidents considered, the extent was set to 20km resulting in 3000x3000 cell matrices, and the ArcGIS feature layers are given below.

To begin, each ArcGIS feature layer is queried from its corresponding URL and parsed into individual features. The raw linear feature set is first filtered to ignore any features that would not appear in the output, a necessity because a single feature may surround but not actually intersect the considered location. The trimmed feature set is then interpolated to match the specified extent so that no gaps appear in the final matrices. After interpolation, if the layer does not contain inaccessibility information such as walking trail or powerline layers, the interpolated features are simply rounded to convert to matrix indices and added to the linear feature matrix. Further, if the layer does contain inaccessibility information, such as river or lake layers might, those locations strictly enclosed by each feature are also interpolated and added to the inaccessibility matrix. The enclosed area is calculated by defining a 2D polygon with the non-interpolated features and checking each point's membership within the polygon. The final step is to merge the ArcGIS linear feature matrix with the elevation-based linear features discussed below.

ArcGIS layers for linear features and inaccessible areas

- Roads: https://carto.nationalmap.gov/arcgis/rest/services/transportation/MapServer/30
- Rivers: https://hydro.nationalmap.gov/arcgis/rest/services/nhd/MapServer/6
- Creeks: https://hydro.nationalmap.gov/arcgis/rest/services/nhd/MapServer/8
- Lakes: https://hydro.nationalmap.gov/arcgis/rest/services/nhd/MapServer/9
- Powerlines: https://services2.arcgis.com/FiaPA4ga0iQKduv3/arcgis/rest/services/US_Electric_Power_Transmission_Lines/FeatureServer
- Railroads: https://carto.nationalmap.gov/arcgis/rest/services/transportation/MapServer/38
- Walking Trails: https://partnerships.nationalmap.gov/arcgis/rest/services/USGSTrails/MapServer/0

Elevation-based linear features

The elevation of the map region is used to find terrain-based linear features like mountain crests or drainages. For each incident, a matrix of elevation with respect to sea level is generated and smoothed to reduce noise, its gradient is computed, and then 1D features of the gradient are found using Canny Edge Detection. To smooth the images, we use the MATLAB function *imgaussfilt*, which filters the elevation matrix, with a 2-D Gaussian smoothing kernel specified by a value of $\sigma = 2$, which is the standard deviation of the Gaussian distribution[1]. To find the gradient of the smoothed elevation, we use the MATLAB function *imgradient* with the Central Difference method[2]. Here, the gradient of each pixel is a weighted difference of its neighboring pixels, such that in the *y*-direction, $dI/dy = (I(y+1) - I(y-1))/2$, where I is the smoothed elevation matrix. Lastly, we find the 1D features by using MATLAB's function *edge* using the Canny detection method[3]. In this approach, the function finds so-called edges by looking for local maxima of the gradient of the input I (the smoothed elevation) using two thresholds to detect strong and weak edges. The range we use is $[0.01, 0.3]$. In having a high and low threshold, the edge function starts with the low sensitivity result from the high threshold and increases it to include connected edge pixels from the higher sensitivity result from the low threshold. In this way, it can fill in the gaps in the detected edges to form smoother lines for the linear features. After obtaining this binary matrix of elevation linear features, we combine it with the ArcGIS linear feature matrix from above.

The full map generation code is available at: https://git.caslab.ece.vt.edu/hlarkin3/ags_grabber

Datasets

The model simulation results are provided as three separate CSV files: 1) *SimulationResults.csv* contains the latitude (N/S) coordinates, the longitude (E/W) coordinates, and the time out of 100 simulation hours of the closest points for each of the 65 incidents, 2) *BehavioralProfiles.csv* contains the 65 incidents' best behavioral profiles consisting of the six strategies: Random Walking (RW), Route Traveling (RT), Direction Traveling (DT), Staying Put (SP), View Enhancing (VE), and Backtracking (BT), and 3) *IPPandFindLocation.csv* contains the 65 incidents' latitude (N/S) and longitude (E/W) coordinates for the IPP and find location.

References:

1. MathWorks, "imgaussfilt," 2021. [Online]. Available: https://www.mathworks.com/help/images/ref/imgaussfilt.html.
2. MathWorks, "imgradient," 2021. [Online]. Available: https://www.mathworks.com/help/images/ref/imgradient.html.
3. MathWorks, "edge," 2021. [Online]. Available: https://www.mathworks.com/help/images/ref/edge.html.
